# Smac mimetic-induced upregulation of interferon-*β* sensitizes glioblastoma to temozolomide-induced cell death

**DOI:** 10.1038/cddis.2015.235

**Published:** 2015-09-17

**Authors:** V Marschall, S Fulda

**Affiliations:** 1 Institute for Experimental Cancer Research in Pediatrics, Goethe-University, Komturstrasse 3a, Frankfurt 60528, Germany; 2 German Cancer Consortium (DKTK), Heidelberg, Germany; 3 German Cancer Research Center (DKFZ), Heidelberg, Germany

## Abstract

Inhibitor of apoptosis (IAP) proteins are frequently expressed at high levels in cancer cells and represent attractive therapeutic targets. We previously reported that the Smac (second mitochondria-derived activator of caspases) mimetic BV6, which antagonizes IAP proteins, sensitizes glioblastoma cells to temozolomide (TMZ)-induced cell death in a nuclear factor-*κ*B (NF-*κ*B)-dependent manner. However, BV6-induced NF-*κ*B target genes responsible for this synergistic interaction have remained elusive. Using whole-genome gene expression profiling, we here identify BV6-stimulated, NF-*κ*B-dependent transcriptional upregulation of interferon-*β* (IFN*β*) and IFN-mediated proapoptotic signaling as critical events that mediate BV6/TMZ-induced apoptosis. Knockdown of IFN*β* significantly rescues cells from BV6/TMZ-induced cell death. Similarly, silencing of the corresponding receptor IFN*α*/*β* receptor (IFNAR) confers a significant protection against apoptosis, demonstrating that IFN*β* and IFN signaling are required for BV6/TMZ-mediated cell death. Moreover, BV6 and TMZ cooperate to transcriptionally upregulate the proapoptotic B-cell lymphoma 2 family proteins Bax (Bcl-2-associated X protein) or Puma (p53-upregulated modulator of apoptosis). Knockdown of Bax or Puma significantly decreases BV6/TMZ-induced apoptosis, showing that both proteins are necessary for apoptosis. By identifying IFN*β* as a key mediator of BV6/TMZ-induced apoptosis, our study provides novel insights into the underlying molecular mechanisms of Smac mimetic-mediated chemosensitization with important implications for the development of novel treatment strategies for glioblastoma.

Glioblastoma is the most common primary malignant brain tumor and current treatment options include surgical resection, radiation and chemotherapy with the alkylating agent temozolomide (TMZ).^1^ However, despite aggressive treatment regimens, the prognosis of patients suffering from glioblastoma is still very poor,^2^ highlighting the high medical need for novel treatment strategies.

Evasion of programmed cell death is one of the hallmarks of human cancers^3^ and promotes tumorigenesis as well as treatment resistance.^4^ Apoptosis is a common form of programmed cell death that can be engaged via the intrinsic (mitochondrial) or extrinsic (death receptor) pathway.^5^ Activation of the intrinsic pathway is controlled by pro- and antiapoptotic B-cell lymphoma 2 (Bcl-2) family protein, including Bcl-2 family proteins such as p53-upregulated modulator of apoptosis (Puma) or Bcl-2-associated X protein (Bax). Following engagement of the mitochondrial pathway, mitochondrial intermembrane space proteins are released into the cytosol, including second mitochondria-derived activator of caspases (Smac).^6^ Smac binds to and neutralizes Inhibitor of Apoptosis (IAP) proteins, a family of antiapoptotic proteins, thereby promoting activation of caspases and apoptosis.^7^ In addition, binding of Smac to IAP proteins that harbor a Really Interesting New Gene (RING) domain with E3 ligase activity triggers their autoubiquitination and proteasomal degradation, which in turn leads to stabilization of nuclear factor-*κ*B (NF-*κ*B)-inducing kinase (NIK) and activation of noncanonical NF-*κ*B signaling.^8, 9^

IAP proteins are expressed at high levels in various cancers and represent attractive targets for therapeutic intervention.^7^ BV6 is a synthetically designed Smac mimetic that mimics the N-terminal part of endogenous Smac protein.^8^ We previously reported that Smac mimetics such as BV6 sensitize glioblastoma cells to chemotherapy- or *γ*-irradiation-induced apoptosis in an NF-*κ*B-dependent manner.^10, 11, 12^ Although BV6-stimulated NF-*κ*B activation was demonstrated to be critically required for Smac mimetic-mediated sensitization of glioblastoma cells towards TMZ, the proapoptotic NF-*κ*B-regulated target genes that mediate this chemosensitization have so far remained elusive.^12^ While tumor necrosis factor *α* (TNFα), a prototypic NF-*κ*B target gene, was shown to mediate apoptosis via an autocrine/paracrine loop upon treatment with Smac mimetic alone^8, 9, 13^ or in combination with anticancer drugs in different carcinoma cell lines,^14^ TNF*α* was found to be largely dispensable for BV6/TMZ-induced apoptosis in glioblastoma cells.^12^ In the present study, we therefore aimed at discovering novel NF-*κ*B-dependent factors that are required for the cooperative anticancer activity of BV6 and TMZ, the prototypic chemotherapeutic agent used for the treatment of glioblastoma.

## Results

### BV6/TMZ cotreatment upregulates IFN-responsive genes before cell death induction

**Figure 1:**
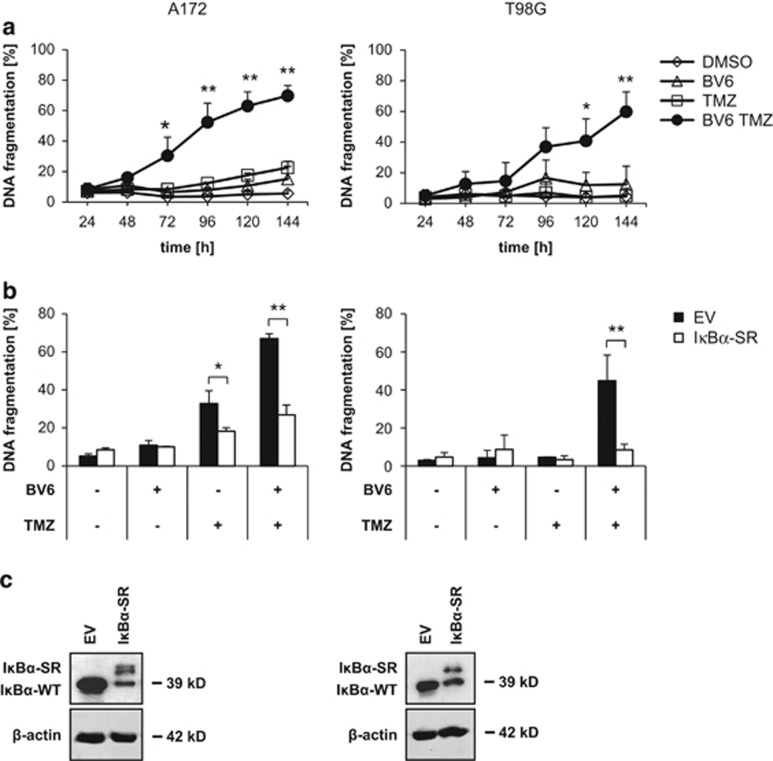
BV6/TMZ cotreatment upregulates IFN-responsive genes. (**a**) A172 cells (left) or T98G cells (right) were treated for indicated times with 100 *μ*M TMZ and/or 2 *μ*M BV6 (A172) or 4 *μ*M BV6 (T98G) or dimethyl sulfoxide (DMSO). Apoptosis was determined by fluorescence-activated cell sorting (FACS) analysis of DNA fragmentation of PI-stained nuclei. Mean values +S.D. of three to four independent experiments performed in triplicate are shown; *P<0.05; **P<0.01 compared with all other settings. (**b**) A172 cells (left) or T98G cells (right) stably expressing I*κ*B*α*-SR or EV were treated for 120 h with 100 *μ*M TMZ and/or 2 *μ*M BV6 (A172) or 4 *μ*M BV6 (T98G) or DMSO. Apoptosis was determined by FACS analysis of DNA fragmentation of PI-stained nuclei. Mean values +S.D. of three independent experiments performed in triplicate are shown; *P<0.05; **P<0.01 compared with all other settings. (**c**) A172 cells (left) or T98G cells (right) stably expressing I*κ*B*α*-SR or EV were analyzed for I*κ*B*α* expression levels by western blotting. Expression of *β*-actin served as a loading control. A representative experiment of two independent experiments is shown

Initially, we assessed cell death upon treatment with the chemotherapeutic agent TMZ and the Smac mimetic BV6 using the glioblastoma cell lines A172 and T98G to confirm that Smac mimetic enhances TMZ-induced apoptosis, as we reported previously.^12^ Indeed, determination of DNA fragmentation as a marker of apoptosis demonstrated that BV6 sensitizes glioblastoma cells to TMZ-induced apoptotic cell death (Figure 1a and Supplementary Figure 1). As Smac mimetics have been described to activate NF-*κ*B signaling, we stably overexpressed dominant-negative I*κ*B*α*-superrepressor (I*κ*B*α*-SR) to block NF-*κ*B signaling^15^ (Figure 1c). Remarkably, inhibition of NF-*κ*B by I*κ*B*α*-SR almost completely rescued cells from BV6/TMZ-induced apoptosis (Figure 1b), underlining the proapoptotic role of NF-*κ*B signaling in this context. To further investigate which BV6-induced NF-*κ*B target genes are responsible for proapoptotic signaling upon BV6/TMZ cotreatment, we performed whole-genome expression profiling using an expression bead chip hybridization assay. Whole-genome expression data were ranked according to fold upregulation comparing A172 glioblastoma cells expressing empty vector (EV) with and without BV6/TMZ treatment. Expression data showing upregulation in A172 glioblastoma cells expressing I*κ*B*α*-SR served as control to identify background expression of non-NF-*κ*B-regulated genes. BV6-treated cells showed a similar expression pattern as BV6/TMZ cotreated cells (data not shown). Interestingly, gene set enrichment analysis (GSEA) revealed upregulation of interferon (IFN)-responsive genes after BV6/TMZ treatment (Table 1 and Supplementary Figure 2). These results demonstrate that BV6/TMZ treatment upregulates IFN-responsive genes in an NF-*κ*B-dependent manner.

### BV6-mediated upregulation of IFN*β* sensitizes glioblastoma cells to TMZ-induced apoptosis

**Figure 2:**
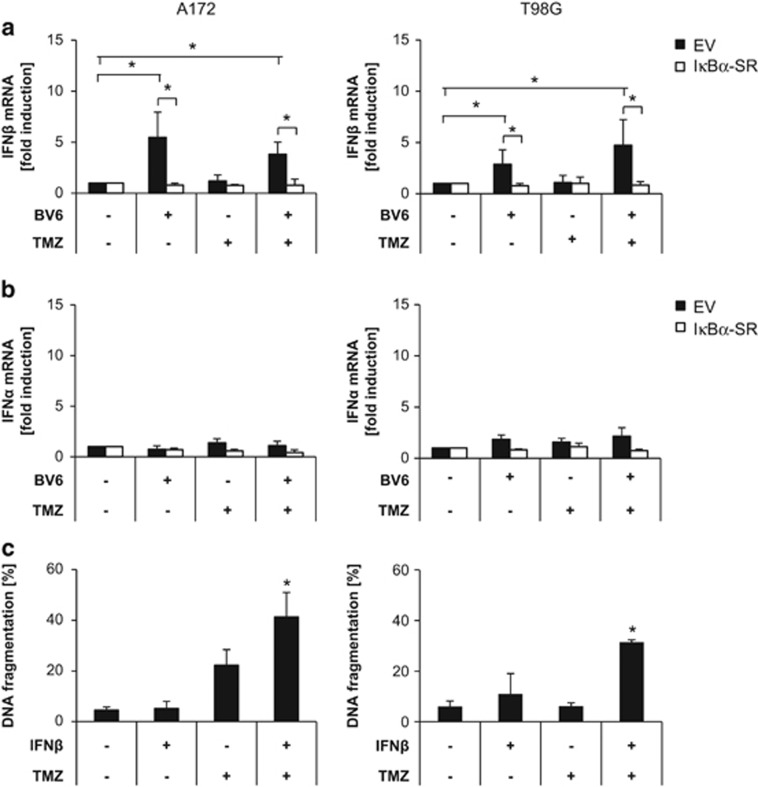
BV6-mediated upregulation of IFN*β* sensitizes glioblastoma cells to TMZ-induced apoptosis. A172 cells (left) or T98G cells (right) stably expressing I*κ*B*α*-SR or EV were treated for 6 h with 100 *μ*M TMZ and/or 2 *μ*M BV6 (A172) or 4 *μ*M BV6 (T98G) or dimethyl sulfoxide (DMSO). IFN*β* (**a**) or IFN*α* (**b**) mRNA levels were analyzed by qRT-PCR, normalized to 28S rRNA expression and fold increase in mRNA levels are shown. Mean values+S.D. of three to four independent experiments performed in duplicate are shown. *P<0.05; **P<0.01. (**c**) A172 cells (left) or T98G cells (right) were treated for 120 h with 100 *μ*M TMZ and/or 1 ng/ml IFN*β* or DMSO. Apoptosis was determined by fluorescence-activated cell sorting (FACS) analysis of DNA fragmentation of PI-stained nuclei. Mean values+S.D. of three independent experiments performed in triplicate are shown; *P<0.05; **P<0.01

Next, we investigated whether IFNs are involved in BV6/TMZ-induced cell death. Type I IFNs such as IFN*α* and IFN*β* have been reported to synergize with TMZ in cell death induction in glioblastoma cells.^16, 17^ Therefore, we analyzed mRNA expression levels of IFN*α* and IFN*β* upon treatment with BV6 and/or TMZ using quantitative real-time-PCR (qRT-PCR) analysis, as IFNs were not represented on the expression bead chip hybridization assay. Interestingly, IFN*β* was upregulated upon BV6 single treatment, as well as upon BV6/TMZ cotreatment (Figure 2a). In addition, BV6-stimulated transcriptional upregulation of IFN*β* was inhibited in I*κ*B*α*-SR-overexpressing cells (Figure 2a), demonstrating that it occurs in an NF-*κ*B-dependent manner. In contrast to IFN*β*, IFN*α* mRNA expression levels remained largely unchanged upon treatment with BV6 and/or TMZ (Figure 2b).

To explore whether IFN*β* acts in concert with TMZ to cause cell death, we treated glioblastoma cells with IFN*β* alone and in combination with TMZ. Intriguingly, IFN*β* significantly increased TMZ-induced cell death in A172 and T98G cells compared to treatment with either agent alone (Figure 2c). In addition to IFN*β*, IFN*α* significantly enhanced TMZ-induced cell death in glioblastoma cells (Supplementary Figure 3). This set of experiments demonstrates that BV6/TMZ induces upregulation of IFN*β* in an NF-*κ*B-dependent manner and that IFN*β* and TMZ cooperate to induce apoptosis in glioblastoma cells.

### IFN*β* is required for BV6/TMZ-induced apoptosis

**Figure 3:**
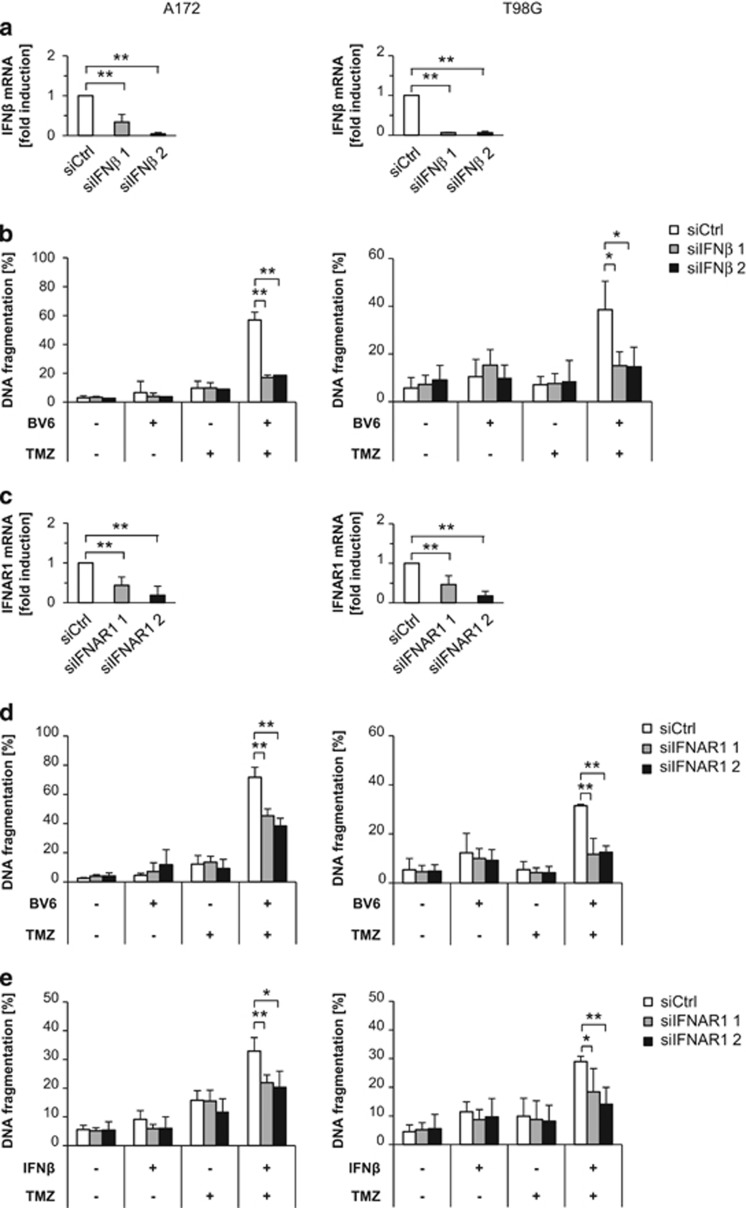
IFN*β* is a crucial mediator of BV6/TMZ-induced cell death. (**a**) A172 cells (left) or T98G cells (right) were transiently transfected with small interfering RNA (siRNA) against IFN*β*. IFN*β* mRNA levels were analyzed after 120 h by qRT-PCR, normalized to 28S rRNA expression and fold increase in mRNA levels are shown. Mean values+S.D. of three independent experiments performed in duplicate are shown. *P<0.05; **P<0.01. (**b**) A172 cells (left) or T98G cells (right) were transiently transfected with siRNA against IFN*β*. Cells were treated for 120 h with 100 *μ*M TMZ and/or 2 *μ*M BV6 (A172) or 4 *μ*M BV6 (T98G) or dimethyl sulfoxide (DMSO). Apoptosis was determined by fluorescence-activated cell sorting (FACS) analysis of DNA fragmentation of PI-stained nuclei. Mean values+S.D. of three to four independent experiments performed in triplicate are shown; *P<0.05; **P<0.01. (**c**) A172 cells (left) or T98G cells (right) were transiently transfected with siRNA against IFNAR1. IFNAR1 mRNA levels were analyzed after 120 h by qRT-PCR, normalized to 28S rRNA expression and fold increase in mRNA levels are shown. Mean values+S.D. of three to four independent experiments performed in duplicate are shown. *P<0.05; **P<0.01. (**d**) A172 cells (left) or T98G cells (right) were transiently transfected with siRNA against IFNAR1. Cells were treated for 120 h with 100 *μ*M TMZ and/or 2 *μ*M BV6 (A172) or 4 *μ*M BV6 (T98G) or DMSO. Apoptosis was determined by FACS analysis of DNA fragmentation of PI-stained nuclei. Mean values+S.D. of three independent experiments performed in triplicate are shown; *P<0.05; **P<0.01. (**e**) A172 cells (left) or T98G cells (right) were transiently transfected with siRNA against IFNAR1. Cells were treated for 120 h with 100 *μ*M TMZ and/or 1 ng/ml IFN*β* or DMSO. Apoptosis was determined by FACS analysis of DNA fragmentation of PI-stained nuclei. Mean values+S.D. of four independent experiments performed in triplicate are shown; *P<0.05; **P<0.01

To examine whether IFN*β* is required for BV6/TMZ-induced cell death, we created IFN*β*-knockdown cells (Figure 3a). Remarkably, silencing of IFN*β* significantly inhibited BV6/TMZ-mediated cell death (Figure 3b). Type I IFNs such as IFN*α* and IFN*β* bind to a transmembrane receptor termed IFN*α*/*β* receptor (IFNAR) and thereby induce transcriptional activation of IFN-stimulated genes (ISGs).^18^ To examine whether IFNAR signaling is involved in BV6/TMZ-induced cell death, we generated IFNAR1-knockdown cells (Figure 3c). Silencing of IFNAR1 significantly reduced BV6/TMZ-mediated cell death (Figure 3d). In addition, knockdown of IFNAR1 rescued cells from IFN*β*/TMZ-induced cell death (Figure 3e). Taken together, this set of experiments demonstrates that IFN*β* has an important role in mediating BV6/TMZ-induced cell death.

### BV6/TMZ-induced apoptosis is mediated by cooperative upregulation of Puma and Bax

**Figure 4:**
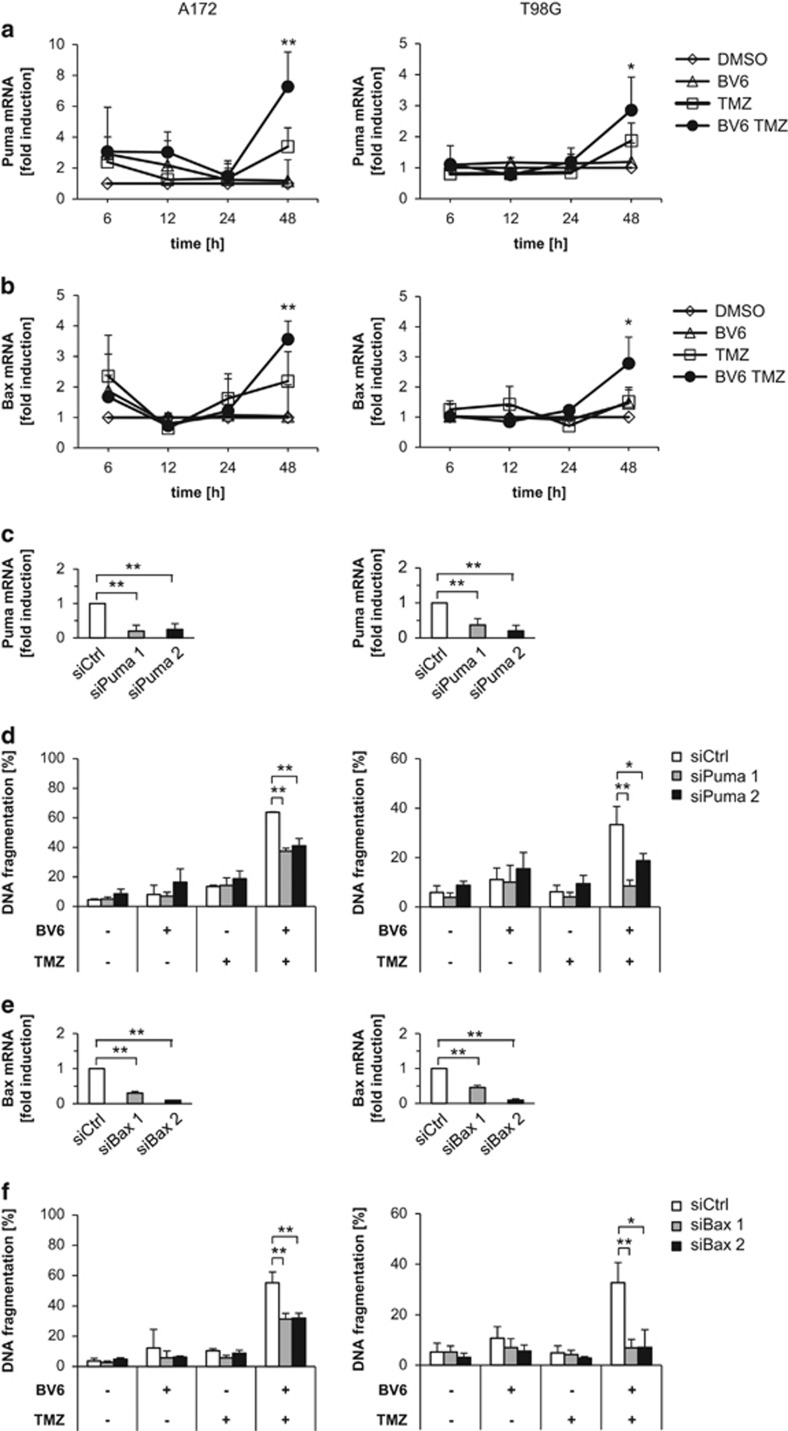
BV6/TMZ-induced apoptosis is mediated by the upregulation of Puma and Bax. A172 cells (left) or T98G cells (right) were treated for indicated times with 100 *μ*M TMZ and/or 2 *μ*M BV6 (A172) or 4 *μ*M BV6 (T98G) or dimethyl sulfoxide (DMSO). Puma (**a**) or Bax (**b**) mRNA levels were analyzed by qRT-PCR, normalized to 28S rRNA expression and fold increase in mRNA levels are shown. Mean values+S.D. of three independent experiments performed in duplicate are shown. *P<0.05; **P<0.01 compared with DMSO control. (**c**) A172 cells (left) or T98G cells (right) were transiently transfected with small interfering RNA (siRNA) against Puma. Puma mRNA levels were analyzed after 120 h by qRT-PCR, normalized to 28S rRNA expression and fold increase in mRNA levels are shown. Mean values+S.D. of three independent experiments performed in duplicate are shown. *P<0.05; **P<0.01 compared with all other settings, if not indicated otherwise. (**d**) A172 cells (left) or T98G cells (right) were transiently transfected with siRNA against Puma. Cells were treated for 120 h with 100 *μ*M TMZ and/or 2 *μ*M BV6 (A172) or 4 *μ*M BV6 (T98G) or DMSO. Apoptosis was determined by fluorescence-activated cell sorting (FACS) analysis of DNA fragmentation of PI-stained nuclei. Mean values+S.D. of three to four independent experiments performed in triplicate are shown; *P<0.05; **P<0.01. (**e**) A172 cells (left) or T98G cells (right) were transiently transfected with siRNA against Bax. Bax mRNA levels were analyzed after 120 h by qRT-PCR, normalized to 28 S rRNA expression and fold increase in mRNA levels are shown. Mean values+S.D. of three independent experiments performed in duplicate are shown. *P<0.05; **P<0.01. (**f**) A172 cells (left) or T98G cells (right) were transiently transfected with siRNA against Bax. Cells were treated for 120 h with 100 *μ*M TMZ and/or 2 *μ*M BV6 (A172) or 4 *μ*M BV6 (T98G) or DMSO. Apoptosis was determined by FACS analysis of DNA fragmentation of PI-stained nuclei. Mean values+S.D. of three independent experiments performed in triplicate are shown; *P<0.05; **P<0.01

ISGs have been described to mediate IFN-induced apoptosis via upregulation of proapoptotic proteins, including proteins of the mitochondrial-dependent cell death pathway.^19^ To link BV6/TMZ-mediated activation of IFN signaling to activation of the mitochondrial apoptotic pathway, we analyzed the expression levels of various proapoptotic Bcl-2 family members (Supplementary Figure 4a). BV6/TMZ cotreatment significantly upregulated mRNA levels of Puma and Bax, whereas no consistent upregulation was observed for Bak (Bcl-2 homologous antagonist/killer), Noxa, Bim (Bcl-2-interacting mediator of cell death), Bid (BH3-interacting domain death agonist) and Bmf (Bcl-2-modifying factor; Figures 4a and b and Supplementary Figure 4a). Also, Puma and Bax were upregulated on the protein level upon BV6/TMZ treatment (Supplementary Figure 4b). To determine whether Puma and Bax are required of BV6/TMZ-induced cell death, we created Puma- or Bax-knockdown cells (Figures 4c and e). Interestingly, knockdown of either Puma or Bax significantly reduced BV6/TMZ-mediated cell death (Figures 4d and f). These results demonstrate that Puma and Bax contribute to BV6/TMZ-induced cell death.

## Discussion

We previously reported NF-*κ*B-dependent sensitization of glioblastoma cells to TMZ-induced apoptosis by the Smac mimetic BV6 as a novel approach to enhance the efficacy of conventional chemotherapy in glioblastoma.^12^ However, the proapoptotic NF-*κ*B target genes mediating this synergistic interaction have so far remained elusive, as autocrine/paracrine TNF*α*/TNF receptor 1 (TNFR1) signaling turned out to be largely dispensable.^12^ In the present study, we identify Smac mimetic-stimulated, NF-*κ*B-dependent upregulation of IFN*β* and IFN-mediated proapoptotic signaling as critical events that mediate BV6/TMZ-induced apoptosis (Supplementary Figure 5). This conclusion is based on the following lines of evidence:

First, treatment with BV6 alone or in combination with TMZ triggers transcriptional upregulation of IFN*β* in an NF-*κ*B-dependent manner, as this increase in IFN*β* mRNA levels is blocked by I*κ*B*α*-SR-mediated inhibition of NF-*κ*B. In addition, gene expression profiling shows an NF-*κ*B-dependent upregulation of ISGs upon BV6/TMZ treatment. Second, BV6-induced upregulation of IFN*β*- and IFN-mediated signaling are required for the induction of apoptosis, as genetic silencing of either IFN*β* or its corresponding receptor IFNAR significantly reduces BV6/TMZ-induced apoptosis. The notion that BV6-stimulated upregulation of IFN*β* promotes TMZ-induced apoptosis is further underscored by data showing that exogenous supply of IFN*β* cooperates with TMZ to trigger apoptosis in glioblastoma cells. Third, we show that IFN*β* and TMZ cooperate to upregulate the proapoptotic Bcl-2 family proteins Puma and Bax, which both contribute to BV6/TMZ-induced apoptosis, as knockdown of Bax or Puma significantly rescues cells from BV6/TMZ-induced apoptosis. Taken together, this identification of Smac mimetic-stimulated, NF-*κ*B-dependent upregulation of IFN*β* and engagement of proapoptotic IFN signaling pathways provides novel insights into the molecular mechanisms that are responsible for Smac mimetic-mediated sensitization of glioblastoma cells to TMZ-induced cell death.

In the present study, we identify IFN*β* as a key mediator of BV6/TMZ-induced cell death that is transcriptionally upregulated in an NF-*κ*B-dependent manner upon treatment with the Smac mimetic BV6. Whether or not this increase in IFN*β* is directly mediated via NF-*κ*B transcription factors^20^ or indirectly mediated via NF-*κ*B-dependent upregulation or activation of transcription factors regulating IFN*β*^21^ remains to be investigated in future studies. It is interesting to note that type I IFNs such as IFN*β* or IFN*α* have recently been reported to act in concert with TMZ to trigger cell death in glioblastoma cells, although the mechanisms responsible for this cooperative effect have so far remained elusive.^16, 17^ In contrast to the critical role of IFN*β* for BV6/TMZ-induced apoptosis that we discovered in the current study, we previously reported that TNF*α*, another prototypic NF-*κ*B target gene, is largely dispensable for BV6/TMZ-induced apoptosis, as addition of the TNF*α*-blocking antibody Enbrel or TNFR1 knockdown failed to rescue apoptosis upon combination treatment.^12^

Although our study demonstrates for the first time that the Smac mimetic BV6 can transcriptionally induce IFN*β* as an important mediator of Smac mimetic-conferred chemosensitization in glioblastoma cells, IFN signaling has been implicated in the past to foster cell death by Smac mimetics. For example, we recently reported that BV6 synergizes with IFN*α* to trigger apoptosis in acute myeloid leukemia cells.^22^ Of note, BV6 was found in the present study to transcriptionally upregulate IFN*β*, but not IFN*α* in glioblastoma cells, pointing to distinct roles of type I IFNs in this context. Moreover, we identified IFN regulatory factor 1 (IRF1) as a novel dual regulator of Smac mimetic BV6-induced apoptosis and proinflammatory cytokine secretion with impact on the immune response.^23, 24^ Furthermore, Smac mimetics have been described to act in concert with innate immune stimuli such as oncolytic viruses and adjuvants, which stimulate a cytokine storm of TNF*α*, TNF-related apoptosis-inducing ligand and IFN*β*, to trigger cancer cell death.^25^

Induction of cell death by IFN*β* has been described to involve ISGs.^19^ However, little is yet known about which ISGs mediate these apoptotic functions. Transcription factors regulated via IFNs such as IRF1 and IRF3 have been reported to promote upregulation or activation of Puma and Bax.^26, 27^ Puma and Bax were also described as DNA damage-induced target genes that are upregulated by TMZ treatment.^28^ Consistently, we show in the present study that IFN*β* and TMZ cooperate to upregulate Puma and Bax, which both contribute to BV6/TMZ-induced apoptosis, as genetic silencing of either Bax or Puma, two Bcl-2 family proteins known to promote mitochondrial apoptosis, rescues glioblastoma cells from cell death. In line with these findings, we previously reported that cotreatment with BV6/TMZ activates the mitochondrial pathway of apoptosis as demonstrated by the loss of mitochondrial membrane potential and cytochrome *C* release.^12^

Furthermore, context-specific settings have an impact on the regulation of signaling pathways and cellular functions by Smac mimetics, depending, for example, on additional cytotoxic stimuli and/or cell types. We demonstrated that Smac mimetics can exert non-apoptotic functions and can stimulate migration and invasion of glioblastoma cells via activation of NF-*κ*B and TNF*α*/TNFR1 autocrine/paracrine signaling.^23, 24^ In glioblastoma cancer stem-like cells, Smac mimetics at non-toxic concentrations can promote astrocytic differentiation by activating NF-*κ*B.^29^

Smac mimetics are currently evaluated in early clinical trials.^30^ By identifying IFN*β* as a novel Smac mimetic-induced and NF-*κ*B-mediated target gene that has an important role in mediating chemosensitization by Smac mimetic, our findings provide novel mechanistic insights into this combination regimen. Additionally, our present study emphasizes that Smac mimetics are effective sensitizers for TMZ-induced apoptosis in glioblastoma cells with implications for the development of experimental treatment approaches.

## Materials and Methods

### Cell culture and chemicals

The human glioblastoma cell lines A172 and T98G were obtained from the American Type Culture Collection (Manassas, VA, USA) and maintained in DMEM medium (Invitrogen, Karlsruhe, Germany) supplemented with 1% penicillin/streptomycin, 1% sodium pyruvate and 10% fetal calf serum (Invitrogen). For experiments, cells were seeded at 5 × 10^3^ cells/cm^2^. Smac mimetic BV6, which neutralizes XIAP, cIAP1 and cIAP2,^8^ was kindly provided by Genentech Inc. (South San Francisco, CA, USA), TMZ was purchased from Sigma (Taufkirchen, Germany) and recombinant human IFNα and IFN*β* from Biochrom (Berlin, Germany). All chemicals were obtained from Sigma, unless indicated otherwise.

### Determination of apoptosis

Apoptosis was assessed by flow cytometric analysis (FACSCanto II; BD Biosciences, Heidelberg, Germany) of DNA fragmentation of propidium iodide (PI)-stained nuclei as described previously.^31^

### Western blotting

Western blot analysis was performed as described previously^12^ using the following antibodies: anti-IκBα (Cell Signaling, Beverly, MA, USA), anti-Bax (BD Biosciences), anti-Puma (Cell Signaling) and anti-*β*-actin (Sigma). Donkey anti-mouse IgG or donkey anti-rabbit IgG labeled with IRDye infrared dyes were used for fluorescence detection at 680–800 nm (LI-COR Biotechnology, Bad Homburg, Germany).

### Whole-genome gene expression array and GSEA

Gene expression profiling was performed as described previously^32^ using Illumina Whole-Genome Expression Beadchips Human HT12v4 (Illumina, San Diego, CA, USA). Expression data were ranked according to fold upregulation comparing A172 glioblastoma cells expressing EV with and without BV6/TMZ treatment. Expression data showing upregulation with and without BV6/TMZ treatment in A172 glioblastoma cells expressing I*κ*B*α*-SR served as control to identify background expression of non-NF-*κ*B-regulated genes. GSEA was performed using software provided by the Broad Institute website (http://www.broadinstitute.org/gsea/index.jsp).^33^

### Transduction and siRNA transfection

Overexpression of the dominant-negative I*κ*B*α*-SR was performed by retroviral transduction as described previously.^15^ For transient knockdown by siRNA, cells were transfected with 20 nM Silencer Select siRNA (Invitrogen) control siRNA (no. 4390844) or targeting siRNAs (s7188 and s7189 for IFN*β*, s782 and s784 for IFNAR1, s1888 and s1890 for Bax, s25840 and s25842 for Puma) using Neon Transfection System (Invitrogen) according to the manufacturer's instructions.

### qRT-PCR analysis

Total RNA extraction and qRT-PCR analysis was performed as described previously^32^ using 7900HT Fast Real-Time PCR System (Applied Biosystems, Darmstadt, Germany). The following primers were used: 28 S (forward, 5′-TTGAAAATCCGGGGGAGAG-3′ reverse, 5′-ACATTGTTCCAACATGCCAG-3′), IFNAR1 (forward, 5′-TCCAGTACATTGTATAAAGACCACAGT-3′ reverse, 5′-GTTCTGATTTTGGACACTGACTTC-3′), Puma (forward, 5′-GACCTCAACGCACAGTACGA-3′; reverse, 5′-GAGATTGTACAGGACCCTCCA-3′), Bax (forward, 5′-AGCAAACTGGTGCTCAAGG-3′ reverse, 5′-TCTTGGATCCAGCCCAAC-3′), Bak (forward, 5′-CCTGCCCTCTGCTTCTGA-3′ reverse, 5′-CTGCTGATGGCGGTAAAAA-3′), Noxa (forward, 5′-GGAGATGCCTGGGAAGAAG-3′ reverse, 5′-CCTGAGTTGAGTAGCACACTCG-3′), Bid (forward, 5′-TGCAGCTCAGGAACACCA-3′ reverse, 5′-TCTCCATGTCTCTAGGGTAGGC-3′), Bim (forward, 5′-CATCGCGGTATTCGGTTC-3′ reverse, 5′-GCTTTGCCATTTGGTCTTTTT-3′), Bmf (forward, 5′-GAGACTCTCTCCTGGAGTCACC-3′ reverse, 5′-CTGGTTGGAACACATCATCCT-3′). Melting curves were plotted to verify the specificity of the amplified products. IFN*α* and IFN*β* mRNA levels were assessed by TaqMan Gene Expression Assay (Life Technologies, Darmstadt, Germany; IFN*α*Hs01077958_s1, IFN*β*Hs00855471_g1) according to the manufacturer's protocol. The relative expression of the target gene transcript and reference gene transcript was calculated as ΔΔC_t_.

### Statistical analysis

Statistical significance was assessed by Student's *t*-test (two-tailed distribution, two-sample unequal variance).

## Figures and Tables

**Table 1:** BV6/TMZ treatment upregulates ISGs

**Enriched gene set**	**ES**
MOSERLE_IFNA_RESPONSE	0.82
SANA_RESPONSE_TO_IFNG_UP	0.81
BROWNE_INTERFERON_RESPONSIVE_GENES	0.77
DER_IFN_GAMMA_RESPONSE_UP	0.76
REACTOME_INTERFERON_GAMMA_SIGNALING	0.74
REACTOME_RIG_I_MDA5_MEDIATED_INDUCTION_OF_ IFN_ALPHA_BETA_PATHWAYS	0.73
BOSCO_INTERFERON_INDUCED_ANTIVIRAL_MODULE	0.72
HECKER_IFNB1_TARGETS	0.71
REACTOME_INTERFERON_ALPHA_BETA_SIGNALING	0.70
DER_IFN_ALPHA_RESPONSE_UP	0.69
DER_IFN_BETA_RESPONSE_UP	0.65
REACTOME_INTERFERON_SIGNALING	0.63

A172 cells stably expressing I*κ*B*α*-SR or EV were treated for 9 h with 100 *μ*M TMZ and/or 2 *μ*M BV6 or DMSO. Whole-genome expression profiling was performed. Genes with similar regulation in A172 cells expressing I*κ*B*α*-SR served as control for background expression of non-NF-*κ*B-stimulated genes. GSEA was performed comparing TMZ/BV6-treated cells to all other settings. The enrichment score (ES) of IFN signaling-mediated gene sets out of the top 100 regulated gene sets upon BV6/TMZ treatment are shown. The false discovery rate for all gene sets shown in the table is <0.0. Mean values of three independent experiments are shown

## Abbreviations

Bak: Bcl-2 homologous antagonist/killer
Bax: Bcl-2-associated X protein
Bcl-2: B-cell lymphoma 2
Bid: BH3-interacting domain death agonist
Bim: Bcl-2-interacting mediator of cell death
Bmf: Bcl-2-modifying factor
IAP: inhibitor of apoptosis
IFN: interferon
IFNAR: IFN*α*/*β* receptor
I*κ*B*α*-SR: I*κ*B*α* superrepressor
IRF: interferon regulatory factor
ISG: IFN-stimulated gene
NF-*κ*B: nuclear factor-*κ*B
NIK: nuclear factor-*κ*B-inducing kinase
Noxa/PMAIP1: phorbol-12-myristate-13-acetate-induced protein 1
Puma: p53-upregulated modulator of apoptosis
RING: Really Interesting New Gene
Smac: second mitochondria-derived activator of caspases
TMZ: temozolomide
TNF*α*: tumor necrosis factor-*α*
TNFR1: tumor necrosis factor-*α* receptor 1
