# Supplementary material for: Smac mimetic-induced upregulation of interferon-β sensitizes glioblastoma to temozolomide-induced cell death
Source: Cell Death Dis. 2015 Sep 17;6(9):e1888–. doi: 10.1038/cddis.2015.235 (PMC4650438; doi:10.1038/cddis.2015.235)
Supplement: Supplementary Figures [file cddis2015235x1.pdf]

**Suppl. Fig. 1**

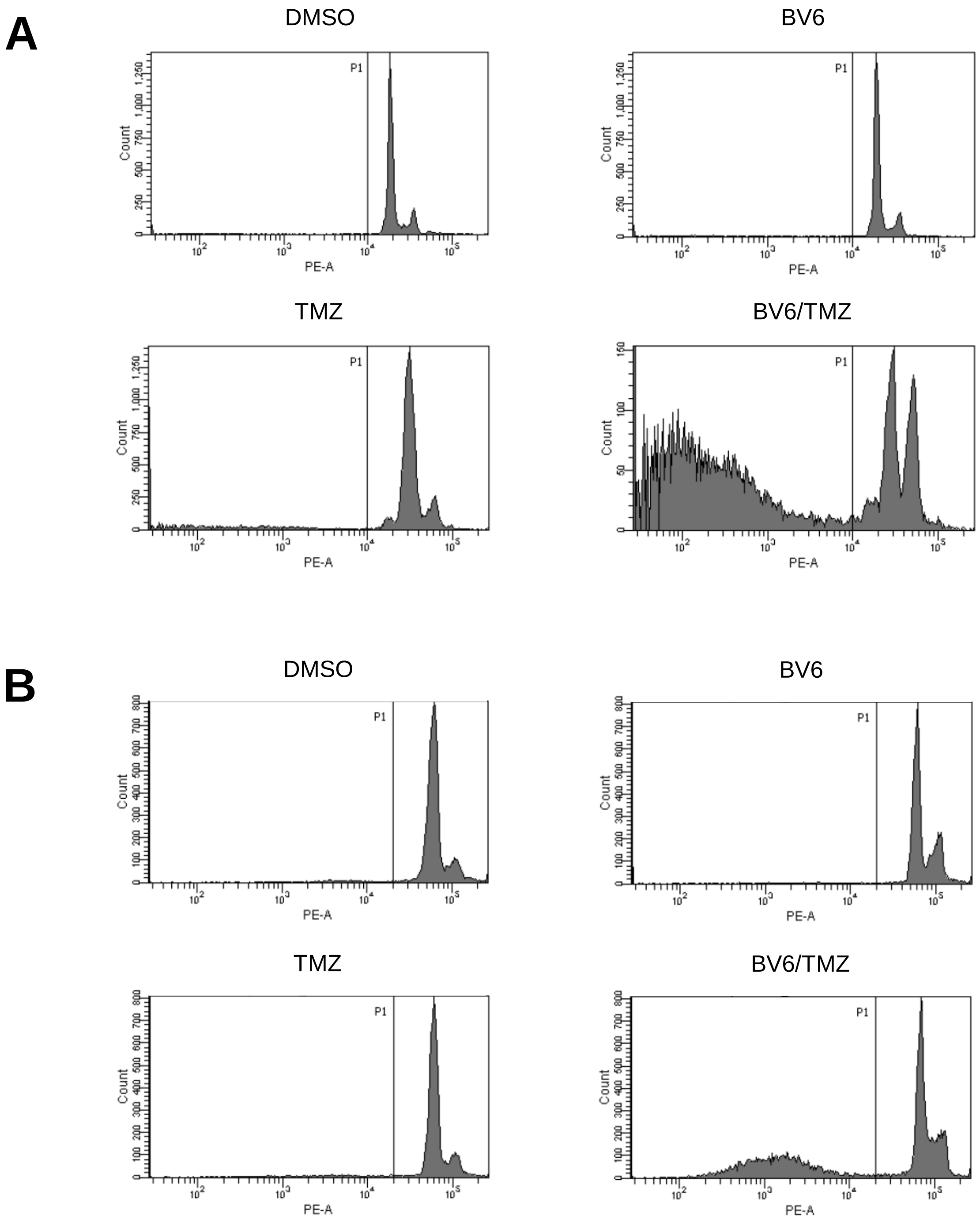

# Suppl. Fig. 2

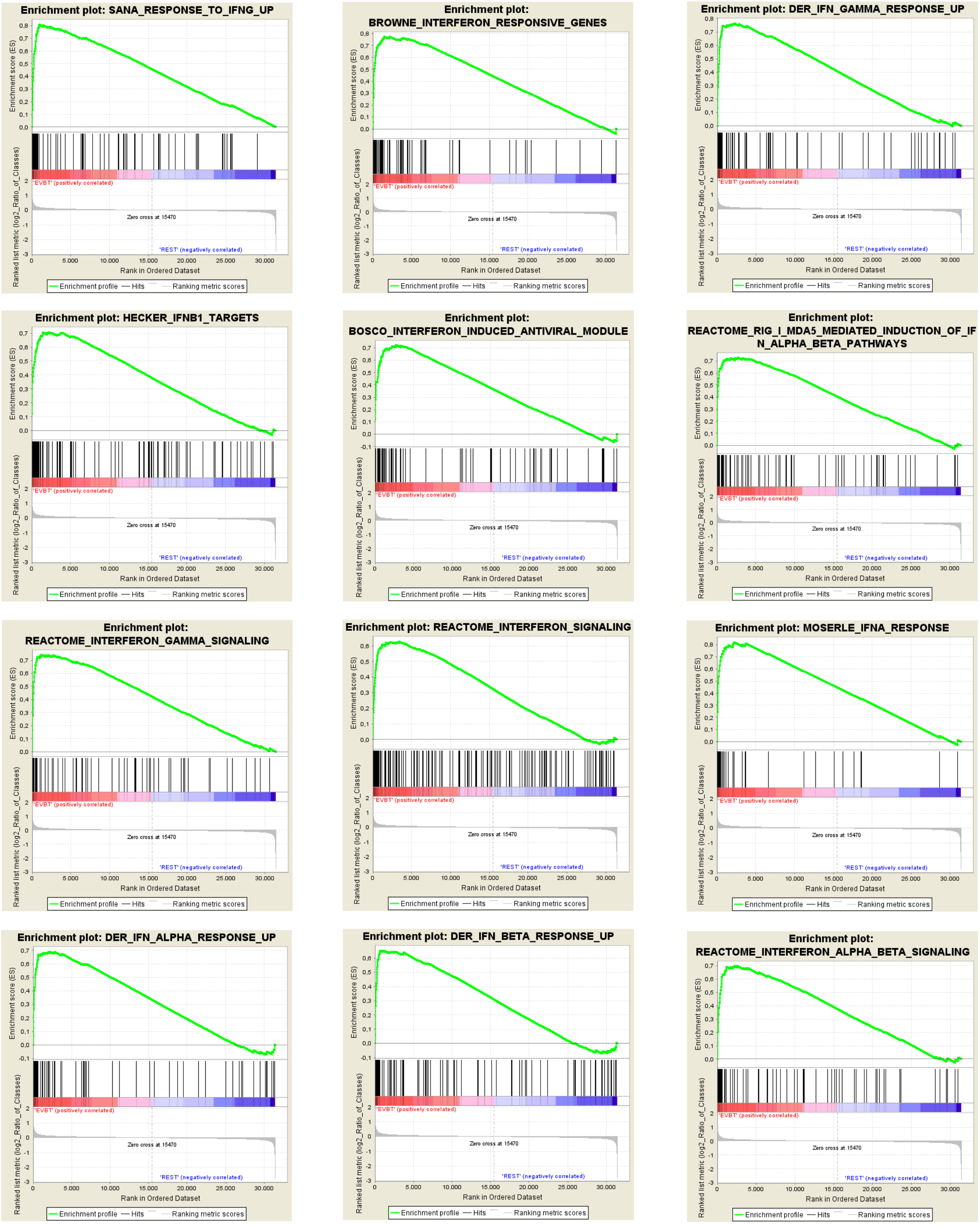

Enrichment plot: SANA_RESPONSE_TO_IFNG_UP
Enrichment plot: BROWNE_INTERFERON_RESPONSIVE_GENES
Enrichment plot: DER_IFN_GAMMA_RESPONSE_UP
Enrichment plot: HECKER_IFNB1_TARGETS
Enrichment plot: BOSCO_INTERFERON_INDUCED_ANTIVIRAL_MODULE
Enrichment plot: REACTOME_RIG_I_MDA5_MEDIATED_INDUCTION_OF_IFN_ALPHA_BETA_PATHWAYS
Enrichment plot: REACTOME_INTERFERON_GAMMA_SIGNALING
Enrichment plot: REACTOME_INTERFERON_SIGNALING
Enrichment plot: MOSERLE_IFNA_RESPONSE
Enrichment plot: DER_IFN_ALPHA_RESPONSE_UP
Enrichment plot: DER_IFN_BETA_RESPONSE_UP
Enrichment plot: REACTOME_INTERFERON_ALPHA_BETA_SIGNALING
Enrichment score (ES)
Ranked list metric (log2_Ratio_of_Classes)
'EVBT' (positively correlated)
Zero cross at 15470
'REST' (negatively correlated)
Rank in Ordered Dataset
Enrichment profile
Hits
Ranking metric scores

**Suppl. Fig. 3**

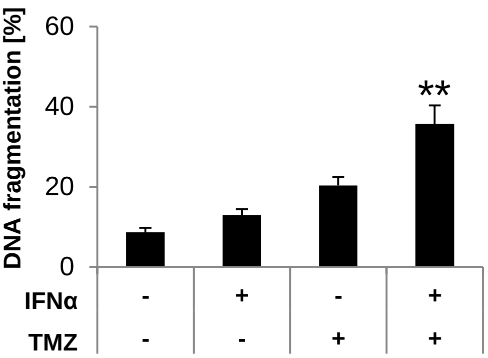

**Suppl. Fig. 4**

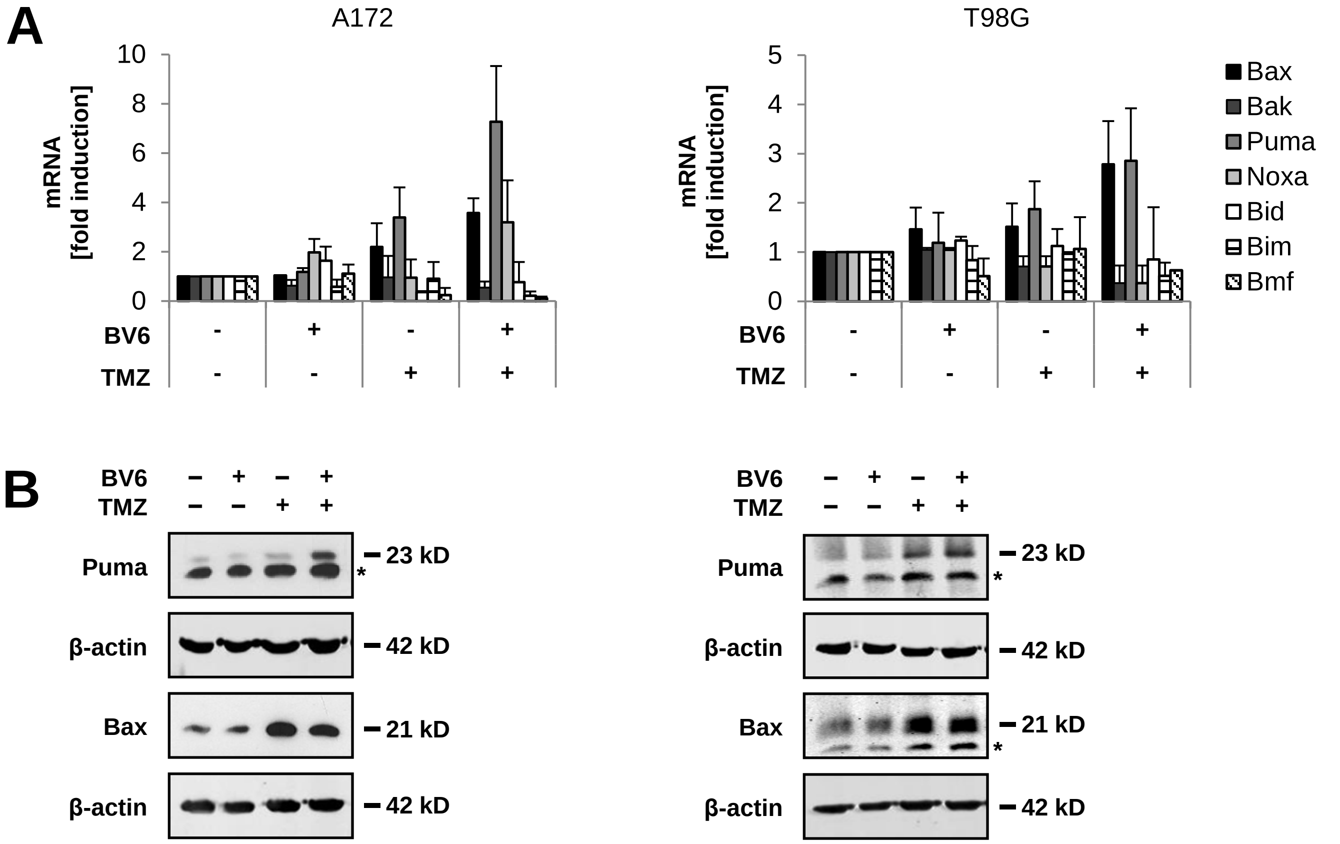
A
A172
T98G
mRNA [fold induction]
Bax
Bak
Puma
Noxa
Bid
Bim
Bmf
BV6
TMZ
B
Puma
β-actin
Bax
23 kD
42 kD
21 kD
*

**Suppl. Fig. 5**

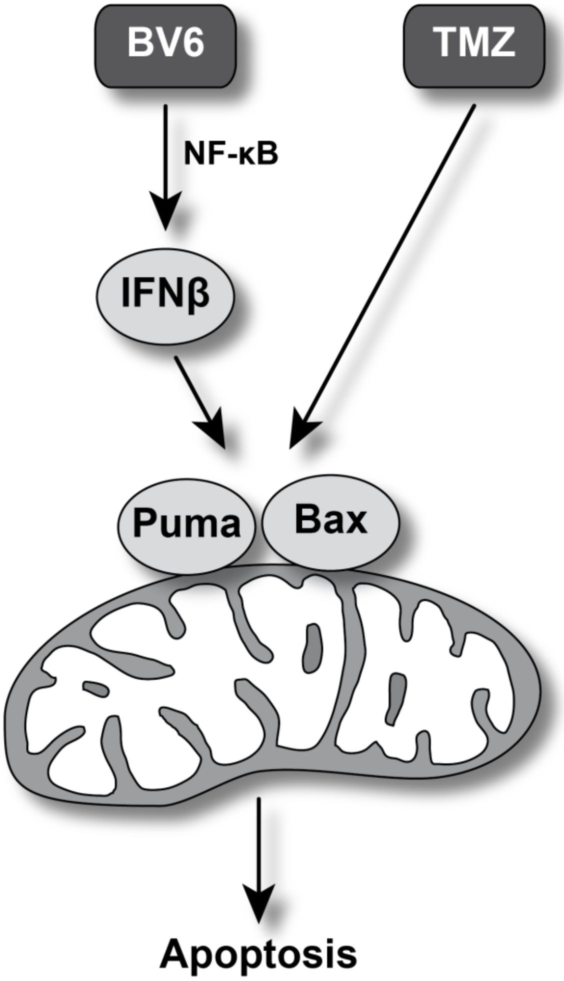

## Supplementary figure legends

**Suppl. Figure 1: BV6 enhances TMZ-induced apoptosis**

A172 cells (a) or T98G cells (b) were treated for 144 hours with 100 μM TMZ and/or or 2 μM BV6 (A172) or 4 μM BV6 (T98G) or DMSO. Apoptosis was determined by FACS analysis of DNA fragmentation of PI-stained nuclei. Representative graphs of three independent experiments performed in triplicate are shown.

**Suppl. Figure 2: BV6/TMZ co-treatment upregulates IFN-responsive genes**

A172 cells stably expressing IκBα-SR or EV were treated for 9 hours with 100 μM TMZ and/or 2 μM BV6 or DMSO. Whole-Genome expression profiling of three independent experiments was performed. Genes with similar regulation in A172 cells expressing IκBα-SR served as control for background expression of non-NF-κB-stimulated genes. GSEA was performed comparing TMZ/BV6-treated cells to all other settings. Enrichment plots of IFN signaling-mediated gene sets out of the top 100 regulated gene sets upon BV6/TMZ-treatment are shown.

**Suppl. Figure 3: IFNα sensitizes glioblastoma cells to TMZ-induced apoptosis**

A172 cells were treated for 120 hours with 100 μM TMZ and/or 1 ng/ml IFNα or DMSO. Apoptosis was determined by FACS analysis of DNA fragmentation of PI-stained nuclei. Mean values + SD of three independent experiments performed in triplicate are shown; *$p<0.05$; **$p<0.01$.

**Suppl. Figure 4: BV6/TMZ-mediated upregulation of Puma and Bax**

(a) A172 cells (left) or T98G cells (right) were treated for 48 hours with 100 μM TMZ and/or 2 μM BV6 (A172) or 4 μM BV6 (T98G) or DMSO. Bax, Bak, Puma, Noxa, Bid, Bim and Bmf mRNA levels were analyzed by qRT-PCR, normalized to 28S rRNA

expression and fold increase in mRNA levels are shown. Mean values + SD of two independent experiments performed in duplicate are shown.

(b) A172 cells (left) or T98G cells (right) were treated for 48 hours with 100 µM TMZ and/or 2 µM BV6 (A172) or 4 µM BV6 (T98G) or DMSO. Expression levels of Bax and Puma were analyzed by western blotting, asterisks indicate unspecific bands. Expression of β-actin served as loading control. A representative experiment of two independent experiments is shown.

**Suppl. Figure 5: BV6-mediated upregulation of IFNβ sensitizes glioblastoma cells to TMZ-induced mitochondrial apoptosis**

Smac mimetic BV6-induced NF-κB-activation promoted transcriptional upregulation of IFNβ. IFNβ and TMZ cooperated to induce apoptosis in glioblastoma cells. Cooperative upregulation of the proapoptotic Bcl-2 family proteins Puma and Bax via BV6/TMZ treatment contributed to BV6/TMZ-induced apoptosis.
